# Risankizumab Efficacy in Synovitis, Acne, Pustulosis, Hyperostosis, and Osteitis (SAPHO) Remission: A Case Report on Rheumatologic and Dermatologic Disease Manifestations with Literature Review

**DOI:** 10.1155/2024/9076852

**Published:** 2024-03-19

**Authors:** Mario Ferraioli, Luigi Fiannacca, Elisabetta Greco, Eneida Cela, Mauro Fatica, Alberto Bergamini, Maria Sole Chimenti

**Affiliations:** Rheumatology, Allergology and Clinical Immunology, Department of Medicina dei Sistemi, University of Rome Tor Vergata, Rome, Italy

## Abstract

SAPHO syndrome is a complex disease that encompasses both inflammatory arthritis and/or osteitis and dermatologic manifestations. It is considered a rare disease, in fact, no clinical trials have been conducted on its therapy and management. Therefore, therapeutic approach is based on small case studies. Here, we described the case of a 63-year-old woman affected by SAPHO syndrome, treated with the selective IL-23p19 antagonist, Risankizumab, after unsuccessful therapies with Methotrexate, Infliximab, Adalimumab, and an allergic reaction to Secukinumab. At the beginning of therapy, in November 2022, the patient presented with arthritis in both knees associated with palmar pustulosis and guttate psoriasis on the trunk. DAPSA score was 24, PtGA 80 mm, PASI score 11.1, and BSA 40%. Thereafter, Risankizumab was started at the standard dosage of 150 mg. At week 24 patient achieved clinical remission, DAPSA score was 8, PtGA was 30 mm, PASI was 1, and BSA 2.5. Patient maintained clinical remission state at the subsequent week 52 evaluation. At the same time, the patient did not report any adverse effects. Health-related quality of life was also assessed at the same time points aforementioned, showing significant improvement. In conclusion, this case report wants to point out the efficacy and safety of Risankizumab in SAPHO syndrome, reporting a sustained disease remission through a 12 months long follow-up period. We can consider IL-23p19 targeted therapy as a novel treatment option for SAPHO—with a high efficacy potential—especially on patients that have already been treated with other biologics.

## 1. Introduction

SAPHO is an acronym for synovitis–acne–pustulosis–hyperostosis–osteitis, a complex and multifaced syndrome first described by Chamot et al. [1]. It is considered a rare disease, with an estimated prevalence of 1/10.000 [2]. Diagnosis is mainly clinical and suspected when an inflammatory arthritis and/or osteitis (particularly if involving the anterior chest wall, sacroiliac joints, or spine) is associated with a neutrophilic or acneiform dermatosis [3]. Khan et al. [4] proposed a set of diagnostic criteria, that even though useful, still lacks validation.

SAPHO syndrome pathogenesis has not been clarified and it probably relies on an intricate interaction between genetic susceptibility, immune dysregulation, and environmental factors [5, 6]. Unlike the spondylarthritis group [7], no clear genetic association has been found [3]. Nevertheless, a possible involvement of both the T helper (Th)-17 pathway and *Cutibacterium acnes* have been suggested. It has been hypothesized that the observed natural killer cells reduction might cause Th-17 cells upregulation that, in turn, might benefit *C. acnes* immune escape [8]. This process might be due to a genetic defect that impairs Forkhead Box O1 (FoxO1) protein [6, 9] resulting in immune activation (with Interleukin (IL) -1, -8, -17A and tumor necrosis factor (TNF)-*α* release), that consequently mediate skin and bone pathological involvement [8, 9]. Moreover, SAPHO classification is still debated. The frequent involvement of the axial skeleton, enthesitis, psoriasis, and inflammatory bowel disease induce its classification among the spondyloarthritis group, but some common features with other autoinflammatory conditions make the latter model more likely.

Regarding therapies, no randomized clinical trials have been conducted and treatments are selected individually for each patient, depending on the clinical phenotype. Nonsteroid anti-inflammatory drugs frequently represent the initial treatment [10]. However, only a small proportion of patients respond, and, in most cases, other therapies are required. Bisphosphonates and classic disease-modifying antirheumatic drugs are often administered as second-line therapies. Even in this case, reported results were not satisfactory [2, 5, 11, 12]. Biologics, on the other hand, have achieved slightly better results [13], even though different depending on the clinical phenotype. Different drugs already in use for the treatment of other rheumatologic diseases [14] have been tried in SAPHO syndrome. TNF-*α* inhibitors appear to have a greater efficacy on bone and joint symptoms (such as osteitis and synovitis) than IL-17 inhibitors. Whereas the latter has shown to better improve dermatologic manifestations [13] and they have also been successfully applied in both psoriasis and psoriatic arthritis [15]. Considering the costimulatory role of IL-23 in Th-17 cells activation and the putative role of IL-17/Th17 in SAPHO syndrome [16], it is possible to speculate that SAPHO patients might also take advantage from IL-23p19 antagonists.

This case report describes a patient with SAPHO syndrome treated with IL-23p19 antagonist Risankizumab. To the best of our knowledge, this is the first known paper reporting Risankizumab efficacy in achieving both rheumatologic and dermatologic disease improvement as well as quality of life (QoL).

## 2. Case Presentation

A 63-year-old European woman with a 20-year history of SAPHO syndrome (palmoplantar pustulosis (PPP), sacroiliitis, and peripheral arthritis) presented in January 2022 to our outpatient clinic of “Policlinico Tor Vergata” (Rome, Italy), reporting arthralgia and swelling in her right knee for approximately 2 months. She is a nonsmoker with a negative family history of any autoimmune or oncologic diseases. Her body mass index was 31.2 kg/m^2^. In 2007, she started methotrexate (10 mg once a week, oral suspension) and, from 2010, Infliximab (5 mg/kg every 6 weeks) at a different rheumatologic clinic. While infliximab was discontinued in 2012 due to achieving satisfactory results on the osteoarticular manifestations, the patient continued methotrexate to control skin eruptions until 2021. Ultrasound with power-Doppler (US-pD) was performed during the visit demonstrating grade 2B-mode synovitis and a grade 2 Doppler signal [17]. Magnetic resonance imaging of the right knee, performed 2 weeks prior, revealed significant effusion, and active synovitis. Blood tests did not show any signs of inflammation. Synovial fluid examination resulted in an inflammatory sterile liquid, with no signs of crystal deposits. Disease activity was assessed as follows [18]: disease activity in psoriatic arthritis (DAPSA) score amounted to 24.3, patient global assessment (PtGA) to 70 mm, psoriasis area and severity index (PASI), and body surface area (BSA) were both negative. Therefore, a diagnosis of SAPHO syndrome was performed. Patient was administered prednisone (7.5 mg) for 2 weeks together with methotrexate (15 mg per week), subcutaneously.

In May 2022, at the first follow-up visit, the patient reported gastrointestinal symptoms (nausea and vomiting) following the methotrexate administration together with persistent skin and joint symptoms. Considering the null improvement with methotrexate (DAPSA = 24.3, PTGA = 65 mm, PASI and BSA = 0), adalimumab therapy (40 mg every other week) was commenced. Thereafter, the patient's symptoms improved, and minimal disease activity [19] was achieved in 8 months.

In August 2022, the patient experienced a recrudescence of both joint and skin manifestations. In fact, the physical examination showed bilateral knee arthritis and the US-pD performed resulted in a grade 2B-mode synovitis and a grade 2 Doppler signal [17] on both knees. Skin examination highlighted multiple pustular lesions on the lower limbs and on both palmar and plantar surfaces, along with guttate psoriatic lesions on the trunk. Again, blood tests did not show any signs of inflammation. Disease activity assessment resulted in a DAPSA score of 24, PtGA of 80 mm, PASI score of 11.1, and a BSA of 40%. Consequently, adalimumab therapy was discontinued, and a therapeutic switch to secukinumab (300 mg every week for 5 weeks then 300 mg every 4 weeks) was decided.

**Figure 1:**
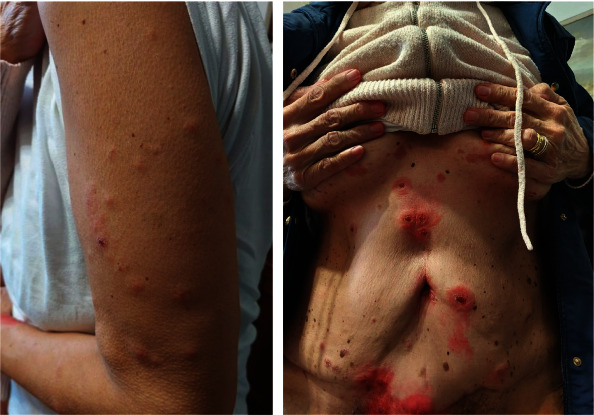
Skin involvement at Sucukinumab therapy withdrawal.

**Figure 2:**
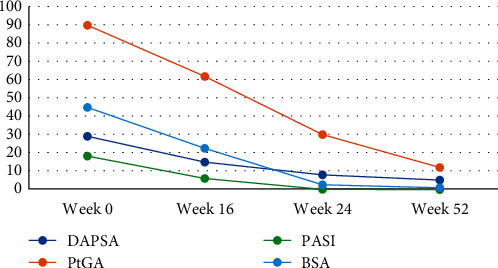
Disease activity indexes during Risankizumab therapy, measured in weeks after therapy initiation. DAPSA, disease activity in psoriatic arthritis; PtGA, patient global assessment; PASI, psoriasis area and severity index; and BSA, body surface area.

In September 2022, following the third administration of secukinumab, the patient reported the appearance of blistering elements with seropurulent content diffused on trunk and thighs. At the same time, the patient reported persistence of previously described skin and joint manifestations. A skin biopsy was performed on one of the new lesions, and it confirmed the clinical suspicion of drug-induced dermatitis, resulting in a diagnosis of *erythema multiforme* (Figure 1). Therefore, Secukinumab was withdrawn and Risankizumab was initiated. The drug was first administered at the end of November 2022, via subcutaneous injection, at the standard dose of 150 mg, followed by a second injection two weeks after, and then one injection every 12 weeks. Disease activity at therapy first administration was as follows: DAPSA score of 29, PtGA of 90 mm, PASI score of 18.3, and a BSA of 45%. After 16 weeks, the patient was reevaluated, cutaneous symptoms had largely improved (PASI = 6, BSA = 22.5%) as well as rheumatologic ones (DAPSA = 15, PtGA = 62). US-pD showed no signs of synovitis on the left knee while on the right one only showed a grade 1B-mode synovitis (grade 0 Doppler signal) [17]. At week 24, patient achieved clinical remission [20], with no US-pD signs of synovitis on both legs. DAPSA score was 8, PtGA was 30, PASI was 1, and BSA 2.5. The last evaluation was performed at 52 weeks, the patient was still in clinical remission (DAPSA = 5, PtGA = 12, PASI = 0, and BSA = 1).  Figure 2 pictures clinical indexes during follow-up. Health-related QoL was also assessed at the same time points aforementioned, through the SF-36 questionnaire [21] (Table 1), showing an improvement in all the domains throughout the entire treatment, the patient did not report any adverse effects.

## 3. Discussion

This case report illustrates the efficacy and safety of Risankizumab therapy on a patient affected by SAPHO syndrome. Overall, the drug had an optimal impact on patient's both dermatological and rheumatological symptoms. In fact, all the disease activity indexes picture a descending curve, and the patient was able to reach disease remission in 6 months of therapy. Additionally, Risankizumab therapy also improved health-related QoL as early as week 16 after treatment start. In fact, the SF-36 questionnaire showed significant improvements in all the domains. Particularly, comparing SF-36 items at therapy start and after 52 weeks, *physical functioning*, *pain*, and *general health* improved of 55%, 42.5%, and 45%, respectively.

Risankizumab is a human monoclonal IgG1 antibody directed against IL-23p19. Its efficacy in SAPHO syndrome was also shown by Flora et al. [22] on a single female patient with prevalent skin manifestations (PPP), treated together with colchicine and oral steroids. Nevertheless, Flora et al. [22] reported few data regarding the joint involvement, and health-related QoL was evaluated only considering skin manifestations. Conversely, presented case has an all-around focus on the effect of Risankizumab on SAPHO syndrome, both considering the rheumatologic and dermatologic aspects of the disease. In fact, treated patients were not assuming any other drugs and a complete joints and skin disease activity evaluation has been reported.

On the other hand, the use of other IL-23p19 antagonists has been previously described in SAPHO syndrome. Mori et al. [23] reported about two patients treated with IL-23p19 inhibitors, without disclosing which of the three available was used nor describing the therapeutic effects. Licata et al. [24] successfully treated one patient with Tildrakizumab, improving their skin manifestations after 4 weeks. On the other hand, authors reported few data on the articular manifestations. Moreover, a Japanese phase III randomized controlled trial of Guselkumab versus placebo in PPP, showed significant improvements in both skin manifestations and health-related QoL [25]. Additionally, a subgroup analyses of the same PPP population [26], highlighted interesting results on those patients with active pustulotic arthro–osteitis—a disease analogous to SAPHO. In fact, subjects treated with Guselkumab showed improvement in magnetic resonance imaging scores of various joints together with improved health-related QoL and decreased CRP levels.

In conclusion, this paper wants to point out the efficacy and safety of Risankizumab in SAPHO syndrome, reporting a sustained disease remission through a long follow-up period. Its strength relies in the detailed assessment conducted on Risankizumab's effects on the main clinical aspects of SAPHO syndrome as well as on QoL. In the author's opinion, IL-23p19 targeted therapy may represent a novel treatment option for SAPHO syndrome—with a high efficacy potential—especially on patients that have already been treated with other biologics.

## Figures and Tables

**Table 1:** Health-related quality of life measured through the SF-36 questionnaire and in weeks after therapy initiation.

	Week 0 (%)	Week 16 (%)	Week 24 (%)	Week 52 (%)
Physical functioning	35	70	80	90
Role limitations due to				
Physical health	50	50	65	45
Emotional problems	66.7	66.7	100	100
Energy/fatigue	45	60	70	75
Emotional well-being	76	64	58	68
Social functioning	37.5	50	50	75
Pain	35	45	55	77.5
General health	20	25	50	65
Health change	25	75	75	75

## Data Availability

Data used to support the findings of this study are available from the corresponding author upon reasonable request.

## Abbreviations

SAPHO: Synovitis–acne–pustulosis–hyperostosis–osteitis
Th: T helper
FoxO1: Forkhead box O1
IL: Interleukin
TNF: Tumor necrosis factor
US-pD: Ultrasound with power-Doppler
PPP: Palmoplantar pustulosis
DAPSA: Disease activity in psoriatic arthritis
PtGA: Patient global assessment
PASI: Psoriasis area and severity index
BSA: Body surface area
QoL: Quality of life.
